# Genomic Surveillance and Phylodynamic Analyses Reveal the Emergence of Novel Mutations and Co-mutation Patterns Within SARS-CoV-2 Variants Prevalent in India

**DOI:** 10.3389/fmicb.2021.703933

**Published:** 2021-07-29

**Authors:** Nupur Biswas, Priyanka Mallick, Sujay Krishna Maity, Debaleena Bhowmik, Arpita Ghosh Mitra, Soumen Saha, Aviral Roy, Partha Chakrabarti, Sandip Paul, Saikat Chakrabarti

**Affiliations:** ^1^Structural Biology and Bioinformatics Division, Council for Scientific and Industrial Research (CSIR) - Indian Institute of Chemical Biology (IICB), Kolkata, India; ^2^Academy of Scientific and Innovative Research (AcSIR), Ghaziabad- 201002, India; ^3^Cell Biology & Physiology Division, Council for Scientific and Industrial Research (CSIR) - Indian Institute of Chemical Biology (IICB), Kolkata, India; ^4^MEDICA Superspecialty Hospital, Kolkata, India

**Keywords:** genome sequencing, India, mutation – genetics, phylodynamic analyses, SARS-CoV-2, COVID-19

## Abstract

Identification of the genomic diversity and the phylodynamic profiles of prevalent variants is critical to understand the evolution and spread of SARS-CoV-2 variants. We performed whole-genome sequencing of 54 SARS-CoV-2 variants collected from COVID-19 patients in Kolkata, West Bengal during August–October 2020. Phylogeographic and phylodynamic analyses were performed using these 54 and other sequences from India and abroad that are available in the GISAID database. We estimated the clade dynamics of the Indian variants and compared the clade-specific mutations and the co-mutation patterns across states and union territories of India over the time course. Frequent mutations and co-mutations observed within the major clades across time periods do not show much overlap, indicating the emergence of newer mutations in the viral population prevailing in the country. Furthermore, we explored the possible association of specific mutations and co-mutations with the infection outcomes manifested in Indian patients.

## Introduction

The corona virus disease or COVID-19 pandemic caused by the severe acute respiratory syndrome corona virus-2 (SARS-CoV-2) has created an unprecedented health and financial crisis throughout the world (McKee and Stuckler, 2020; Ibn-Mohammed et al., 2021). Since the emergence of the outbreak in the Chinese city of Wuhan in late 2019, the COVID-19 disease has spread widely and has caused millions of infections and thousands of deaths throughout the year 2020, and causalities are still continuing at an alarming rate in parts of the globe, especially in India. As of June 2021, the total number of infections reported in India surpasses 29 million, while the active infections are still more than 798,000, causing an overall death toll of more than 383,000 (Ministry of health and family welfare, Government of India, 2021). India has already witnessed two waves of infections. The first wave of infections surfaced in the year 2020. At the end of year 2020, the infection rate was reduced, but it surged again in April 2021, leading to the second wave of infections which caused more fatality compared to the first wave of infections. Similar to global efforts pursued to combat the deadly disease, various measures (Majumdar et al., 2021) have been taken by the Indian clinical and biomedical research community, including vaccine development (Kaur and Gupta, 2020; Ella et al., 2021), clinical trials with repurposed drugs (Charan et al., 2020), convalescent plasma therapy (Bandopadhyay et al., 2021), nationwide serological survey (Naushin et al., 2021), and genetic surveillance *via* genome sequencing of the viral samples extracted from infected individuals (Banu et al., 2020; Maitra et al., 2020; Pattabiraman et al., 2020; Raghav et al., 2020; Saha et al., 2020; Yadav et al., 2020; Biswas et al., 2021; Muttineni et al., 2021; Srivastava et al., 2021).

Several thousands of whole-genome sequences of SARS-CoV-2 from various parts of the country have been sequenced and subsequently deposited in global databases such as GISAID (GISAID, 2021). Multiple works from India have highlighted the genomic diversity and the phylogenetic profiles of the prevalent variants in the country (Banu et al., 2020; Sarkar et al., 2021). In this report, we are focused on the first wave of infections, precisely on the sequences deposited in 2020 (till December 31, 2020). We observed that more than 75% of the SARS-CoV-2 sequences (2,521 out of 3,277 complete genomes from India) were deposited in the latter half of the year 2020. The lower number (146) of deposited sequences during December 2019–March 2020 is understandable as the active cases only started to appear in India in March 2020 only. In order to enrich the viral genome sequence data for the latter half of the year, we sequenced 54 SARS-CoV-2 sequences collected from the state of West Bengal during August 2020–December 2020, making it only the third Indian state apart from Telangana and Maharashtra to deposit more than 50 sequences in that time period. With these sequences, we decided to analyze and understand the spatio-temporal evolutionary dynamics of the pathogen across various states and union territories (UT) of India in the year 2020. We estimated the clade dynamics of the Indian variants, compared the clade-specific mutations, speculated their positive selection, and calculated the co-mutation patterns across states and UTs of India. Furthermore, we explored the possible association of specific mutations/co-occurring mutations with the infection outcome manifested in the patients.

We found that, for Indian sequences GR, GH, and G (GISAID) or 20B and 20A (Nextstrain) (Nextstrain, 2021), the clades were primarily the major prevalent clades in the middle and later half of the year 2020. However, frequent mutations observed within each of the major clades do not show much overlap, especially for the last half of the year 2020, indicating the emergence of new mutations in the viral population prevailing in the country. Interestingly, only 10% of the mutations within the GISAID clades across various Indian states are found to be common. The co-mutations or co-occurrence of mutations within a specific viral variant were investigated, and frequent co-mutation patterns for different Indian states were identified. Finally, associations between a specific mutation and a co-mutation pattern with respect to patient status (deceased, symptomatic, asymptomatic, etc.) have been explored.

## Materials and Methods

### Sample Collection

Ethical clearances were taken from the institutional (CSIR-Indian Indian Institute of Chemical Biology) and hospital (MEDICA Superspecialty Hospital) ethical committees for the present study. The nasopharyngeal/oropharyngeal swabs of COVID-19 patients were collected from August to October 2020. All samples were collected at MEDICA Superspecialty Hospital, Kolkata. The samples were anonymized by removing patient identifiers except gender, age, and collection date. The SARS-CoV-2 nucleic acids were isolated using the MagMax Viral Pathogen Isolation kit from Thermo Fisher in KingFisher Flex automated extractor. The RT-PCR assay was performed using SD Biosensor COVID-19 kit based on Taqman probe chemistry for the detection of SARS-CoV-2 RDRP gene and E gene using reverse transcription in Rotor Gene Q 5 plex HRM system. Samples with Ct values ≤25 were considered for sequencing.

### Viral Whole-Genome Sequencing

Virus genomes were sequenced using ARTIC COVID-19 multiplex PCR primer, version 3, *via* a combination of nanopore sequencing based on MinION sequencer, Oxford Nanopore Technology (ONT), and Illumina HiSeqX (Illumina, 2020). To generate PCR amplicons for nanopore sequencing, Native Barcode Expansion 1–12, protocols (Kits EXP-NBD104) (ONT) were used. RNA extracted from the clinical specimens were converted to cDNA using reverse transcriptase enzyme (Super Script IV First Strand Synthesis Kit, Thermo Fisher Scientific, Waltham, MA) and then purified by using AMPure XP beads. The purified cDNA was then amplified by each of the two ARTIC v3 primer pools which tile the SARS-CoV-2 genome. The amplified product was further subjected to end-repair and barcoded by Native Barcode Expansion kits (Banu et al., 2020; Charan et al., 2020; Kaur and Gupta, 2020; McKee and Stuckler, 2020; Bandopadhyay et al., 2021; Biswas et al., 2021; Ella et al., 2021; Ibn-Mohammed et al., 2021; Majumdar et al., 2021; Ministry of health and family welfare, Government of India, 2021; Naushin et al., 2021; Srivastava et al., 2021) and purified by a concentration of 0.4× of AmpureXP. All samples were then pooled together and ligated with sequencing adapters; the purified samples were finally quantified using Qubit 4.0 Fluorometer (Invitrogen), followed by loading of 15 ng of pooled barcoded material and sequencing on MinION flow cells.

For Illumina sequencing, the samples were sequenced through 2 × 150-bp paired-end in Illumina HiSeqX sequencing system following the standard protocol. QIAseq SARS-CoV-2 Primer Panel (Qiagen, cat. no. 333895) and QIAseq FX DNA Library Kit were used in order to prepare amplicon libraries for viral genome sequencing. The prepared libraries were then pooled and sequenced using Illumina HiSeqX instrument to generate 150-bp paired-end reads.

### Genome Assembly and Sequence Submission in GISAID

Two different methods were implemented for the assembly of long and short reads as obtained from ONT and Illumina sequencing platforms, respectively. All reads from both platforms were checked for their quality using FastQC (Andrews, 2021). In case of long reads, reference-based assembly was performed using the first SARS-CoV-2 strain identified from Wuhan, China [NCBI accession number NC-045512.2 (NC_045512, 2021), which is identical to the GISAID reference sequence EPI_ISL_402124 (GISAID, 2021)] as reference and Minimap2 (Li, 2018) with ONT-specific parameters. In case of short reads, they were first filtered using KneadData (KneadData, 2021) and then assembled by SPAdes (Bankevich et al., 2012) using default parameters. Pilon (Walker et al., 2014) was used for polishing and generation of final consensus sequences. The assembled SARS-CoV-2 genome sequences were checked for frameshifts using Genome Detective online tool (Vilsker et al., 2019), and their depths were calculated using Mosdepth (Pedersen and Quinlan, 2018). All the 54 genome sequences with their associated metadata were uploaded to the GISAID database (Supplementary Table 1).

### Sequence and Mutation Data Collection

We have accessed the protein sequences of SARS-CoV-2 virus collected from different continents from the EpiCoV database of GISAID (GISAID, 2021). The database was searched on 1st January 2021 up to sample collection date December 31, 2020 using the primary key-words “hCoV-19” and “human”. Only complete and high-coverage sequences were considered. Sequences with genomes >29,000 bp were considered complete. Sequences with <1% Ns (undefined bases) were considered as high-coverage sequences. Sequences from different states of India were also accessed and analyzed separately. Additional metadata for the sequences, which include the location of sample collection, age and sex of the patients, clade, lineage, and patient status, were also downloaded upon availability.

We found only 23 sequences collected in 2019, all from Wuhan, China, from where the disease spread. To analyze the evolution of viral clades with time, we divided all sequences in three terms, depending on the date of the collection of samples. “Term1” includes sequences collected till March 2020. “Term2” defines April 2020–July 2020, and “Term3” includes sequences collected from August 2020 to December 2020. Table 1 shows the number of sequences collected from different continents and India in different terms.

**TABLE 1:** Number of sequences deposited in the year 2020.

Continent/country/lab	December 2019–March 2020	April 2020–July 2020	August 2020–December 2020	Total
North America	10,877	21,895	8,583	41,355
South America	433	1,076	308	1,817
Europe	16,198	30,827	86,563	133,588
Africa	180	1,670	1,019	2,869
Asia^a^	3,178	9,189	1,249	13,616
Oceania	2,001	6,604	4,184	12,789
India	146	2,521	610	3,277
IICB^b^	0	0	54	54

*^a^Excluding India.*

*^b^Indian Institute of Chemical Biology submitted 54 out of 55 total sequences deposited in GISAID during August 2020–December 2020.*

For identifying the mutations, GISAID reference sequence EPI_ISL_402124, collected from human sample in Wuhan, China, in December 2019, was considered as reference (Zhou et al., 2020).

### Alignment and Mutation Frequency Analysis

To extract the unique representative sequences and exclude redundant sequences, the CD-HIT (Fu et al., 2012) server was used. The number of CD-HIT runs was kept as one with a sequence identity cutoff of 1.0 (100% identity). It provided clusters of sequences which are 100% identical. The cluster representative sequences, along with the reference sequence, were aligned using Kalign protein sequence alignment tool (Madeira et al., 2019). Python (version 3.4) codes were used for extracting mutations and further analysis. Mutation was considered as frequent when the frequency was calculated with at least 50 sequences (*N*), the frequency was ≥2.5% for *N* ≥ 200, and mutation count was at least five when *N* < 200.

### Mutational and Co-mutational Analysis

For mutational analysis within India, we have chosen states and UTs, which had at least 50 sequences in a given term. For Term2, we found eight states and UTs. Among them, only three states had more than 50 sequences in Term3. Hence, temporal analysis was done for those three states only. We also analyzed how different mutations co-occurred in different states and terms. Network was constructed for each state and UT, showing the co-occurrence between frequent mutations of that state/UT. Cytoscape.js (Shannon et al., 2003) was used to construct the network.

### Patient Status Association Analysis

To correlate disease severity with the mutation and co-mutation pattern, available metadata was analyzed. Based on the available “patient status”, the patients were classified broadly in four categories: deceased, symptomatic, mild, and asymptomatic. A total of 806 sequences deposited from India till December 2020 were used, where 95 were marked as deceased and 631, 49, and 31 were marked as symptomatic, mild, and asymptomatic, respectively. Similarly, for the global association study, 333 (North America) and 817 (Europe) samples, with the status of patients from other continents, were used. Patient status was associated with frequent mutations, co-occurring mutations, and clades. Fisher’s exact test was performed using the following contingency table (Hoffman, 2019) for deceased samples:

**Table:** 

	Mutated	Not-mutated	Total
Not deceased	a	b	a + b
Deceased	c	d	c + d
Total	a + c	b + d	a + b + c + d = *N*
			

where *N* is the total number of sequences. Similar tables were used for other types of categories of the patients (symptomatic, mild, and asymptomatic). The probability of obtaining a given set of result, *p*-value, is provided by a hypergeometric distribution,


(1) $$p=\frac{\left(\begin{array}{c} a+c\\ a \end{array}\right)\,\left(\begin{array}{c} b+d\\ b \end{array}\right)}{\left(\begin{array}{c} N\\ a+b \end{array}\right)}$$

where ${(}_{j}^{i})$ denotes the binomial coefficient of any given variable *i* and *j*.

## Results

### Demographic and Phylogenic Distribution

A total of 54 SARS-CoV-2 samples taken from patients residing in Kolkata and West Bengal were sequenced using whole-genome sequencing approach. The ages of the patients ranged from 6 to 88 years, with a maximum peak (24.07%) in the range of 51–60 years (Supplementary Figure 1A) and with an overall male-to-female ratio of 1.7 against the national ratio of 1.99. For comparison, we have also plotted the age distribution of the Indian patients whose extracted SARS-CoV-2 sequences were deposited in the GISAID database (Supplementary Figure 1A). The quality of the sequencing data represented in the form of depth shows a mean depth of approximately 26035X and 455X for short reads and long reads, respectively (Supplementary Figure 1B), while the coverage for all the assembled genomes was above 99%. The GISAID clade distribution of the 54 sequences is slightly different from the national distribution with majority of O clade representation, and the same is true for Nextstrain clades with a prevalence of 20A clade (Supplementary Figures 1C,D).

### Dynamics of Clade Distribution

**FIGURE 1:**
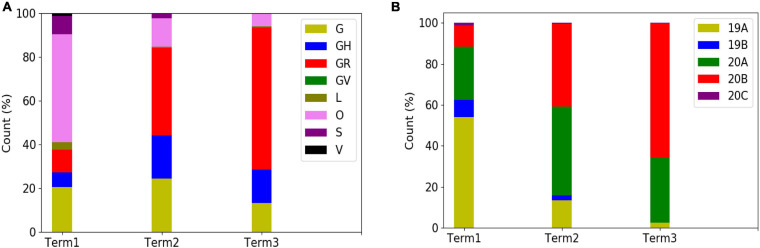
Distribution and dynamics of SARS-CoV-2 clades in India for three different time spans in the year 2020. Distribution of GISAID **(A)** and Nextstrain **(B)** clades across India for three different times spans: “Term1” (December 2019–March 2020), “Term2” (April 2020–July 2020), and “Term3” (August 2020–December 2020), respectively.

In order to explore the evolutionary and mutational dynamics represented in terms of phylogenetic clades, we compared the clade distribution of all the complete and high-quality SARS-CoV-2 sequences (3,277) deposited in GISAID from India until December 31, 2020. The sequences were categorized into three time points (“Term”) based on the date of the collection/submission of the sequences. Figure 1 shows the distribution of the different GISAID and Nextstrain clades in three time points. It is evident that GR clade is prevalent in Term2 and Term3, followed by GH and G clades (Pandit et al., 2021). Similarly, Nextstrain clades 20B and 20A are found to be more prevalent in the latter half of the year 2020. To compare the clade dynamics with respect to SARS-CoV-2 sequences from elsewhere, we have performed a similar analysis with sequences deposited from North America, South America, Europe, Africa, Oceania, and Asia (without Indian sequences) (Supplementary Figure 2). Interestingly, except North America and Europe, all the other continents show a prevalence of GR clade in the second and third term of the year. North America shows a consistent prevalence of GH clade throughout the year, where a massive increase in the numbers of GV clade sequences was observed in Europe during the latter part of 2020. The precise reason behind the observed dominance of a particular clade in a geographical area is yet to be understood completely. It could be influenced by multiple factors including geographical, sociological, physiological, and differences of the host demography and genetics, respectively. However, it is interesting to investigate whether sequences belonging to the major clades observed in India through various time “Terms” harbor similar mutations or not. Hence, we compared the non-clade-defining and “frequent” (frequency ≥2.5% or >5 when *N* ≤ 200) mutations observed within the same clade across the time terms (Supplementary Figure 3). Interestingly, we observed that a significant fraction of the mutations turned out be unique in “Term2” and “Term3”, indicating the accumulation of novel mutations.

### Dynamics of Clade Variation Across Indian States and Union Territories

**FIGURE 2:**
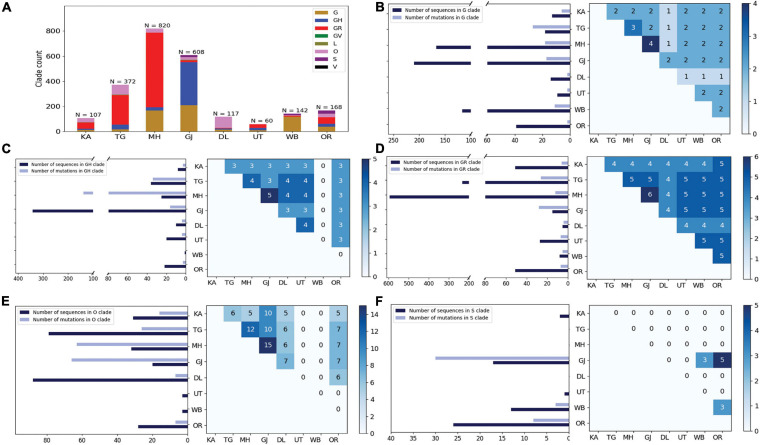
Distribution of GISAID clades within Indian states and union territories and comparison of frequent mutations across them during Term2 (April 2020–July 2020). **(A)** The distribution of GISAID clades in seven Indian states and one union territory (UT) that deposited more than 50 SARS-CoV-2 sequences during April 2020–July 2020. **(B–F)** The number of sequences and frequent mutations of the seven states and one UT and the number of common mutations among them for G, GR, GH, O, and S clades, respectively. KA, Karnataka; TG, Telangana; MH, Maharashtra; GJ, Gujarat; DL, Delhi; UT, Uttarakhand; WB, West Bengal; OR, Odisha.

Curious to see the previous observation, we wanted to investigate whether the mutational variability within clades is more specific to certain geographical locations delineated by the states and UTs of India. “Term1” contains only 146 sequences from India; hence, we compared state-specific mutations by taking SARS-CoV-2 sequences from only “Term2” and “Term3”, respectively. Only seven states and one union territory—Karnataka (KA), Telangana (TG), Maharashtra (MH), Gujarat (GJ), Delhi (DL), Uttarakhand (UT), West Bengal (WB), and Odisha (OR)—contained more than 50 deposited good-quality sequences in GISAID. Figure 2A shows the fraction of various GISAID clade sequences within these states and union territory. MH, GJ, and WB are the top three states representing G clade sequences in “Term2”, whereas GJ produces a significantly higher number of GH clade sequences compared to other states. MH and TG possess the maximum number of GR sequences, followed by KA and OR. Interestingly, DL and TG provide the maximum number of O clade sequences, which are generally less clearly defined (Mercatelli and Giorgi, 2020). The distribution of clade S is underrepresented in these eight states and union territories. A comparison of the frequent mutations across states for the same clade also revealed much more specific mutation than common ones (Figure 2), where overall only 10% of the mutations within the GISAID clades across various Indian states are found to be common (Table 2). In “Term3”, only from three states (MH, TG and WB) were more than 50 sequences deposited, and similar to the observation from “Term2”, very few overlaps of mutations were found to be common across these three states (Supplementary Figure 4 and Table 2).

**TABLE 2:** Specific and common mutations across different states for different clades.

Clades	Unique specific mutations in Term2	Unique common mutations in Term2	Unique specific mutations in Term3	Unique common mutations in Term3
G	68	6	0	2
GH	179	6	5	5
GR	59	7	21	6
GV	0	0	0	0
L	17	5	0	0
O	133	24	0	0
S	30	5	0	0
V	1	0	0	0

### Variation in the Co-occurrence of Mutations (Co-mutations) Across Indian States and Union Territories

**FIGURE 3:**
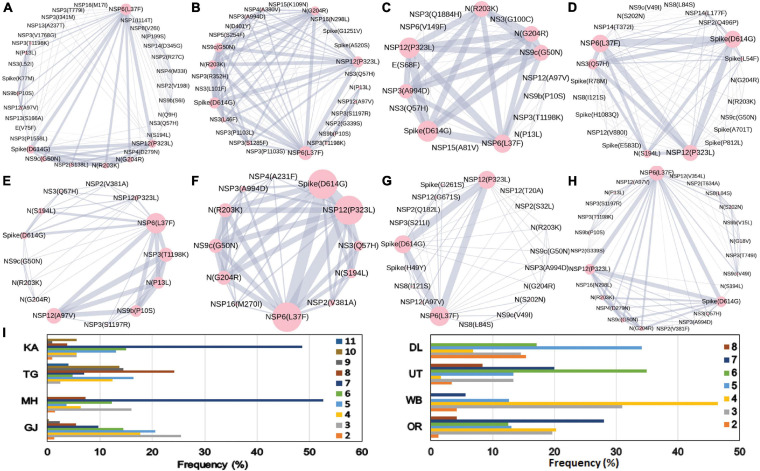
Co-mutation patterns and networks observed within Indian states and union territories (UT) during Term2. The networks of co-mutations for seven states and one UT: **(A)** Karnataka (KA), **(B)** Telangana (TG), **(C)** Maharashtra (MH), **(D)** Gujarat (GJ), **(E)** Delhi (DL), **(F)** Uttarakhand (UT), **(G)** West Bengal (WB), and **(H)** Odisha (OR), where each mutation site is marked as a “node” and the co-mutation is represented as an “edge.” The node size indicates the frequency of mutation, while the edge thickness represents the number of times (sequences) a pair of mutation has co-occurred. **(I)** The bar plot distribution of the actual number of co-mutations within each state and UT.

**FIGURE 4:**
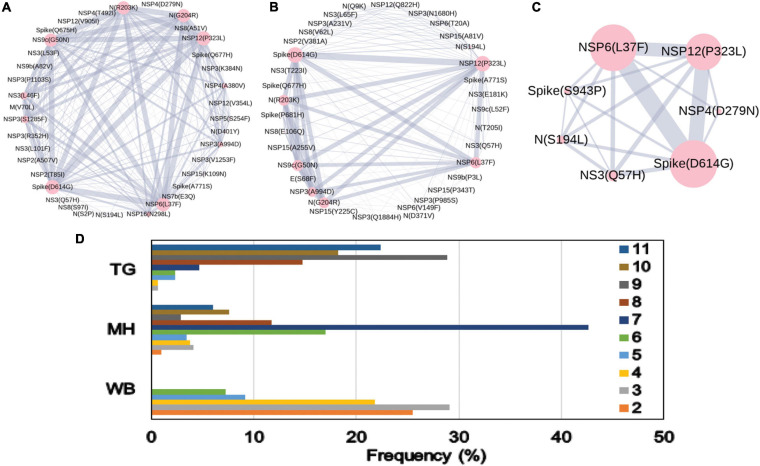
Co-mutation patterns and networks observed within Indian states and union territories during Term3. **(A–C)** The networks of co-mutations for Telangana, Maharashtra, and West Bengal, respectively, where each mutation site is marked as a “node” and the co-mutation is represented as an “edge.” The node size indicates the frequency of mutation, while the edge thickness represents the number of times (sequences) a pair of mutation has co-occurred. **(D)** The bar plot distribution of the actual number of co-mutations within each state.

The combined impact of co-occurrence of mutations within a specific viral variant could be crucial to elicit the infectivity and sustenance of a viral load within the host. Hence, co-mutation patterns within SARS-CoV-2 variants were investigated, and specific/common co-mutations for different Indian states were identified irrespective of clade. Figures 3A–H show the network representation of co-mutation patterns among the most frequent mutations, where each node represents a mutation site and the edge denotes a co-occurrence between a pair of mutant sites. Edge thickness is proportional to the number of co-occurrence, while node size is to the frequency of mutation. Any two nodes (sites) were considered to be co-mutated if the co-mutation pair is represented in ≥2.5% of the population size or >5 when the total number of sequences in the particular category is less than 200. Figure 3I show the frequency distribution of the number of co-mutating sites for viral genomes from different states and union territories. It is evident that, for most of these states and UT, a large fraction of the sequences harbor five or more co-mutations. Although the mutations include the clade-defining mutations [e.g., G: Spike(D614G), GH: Spike(D614G) and NS3(Q57H), GR: Spike(D614G) and N(G204R), GV: Spike(D614G) and Spike(A222V), S: NS8(L84S)], a large number of non-clade-defining mutations are found to co-occur within SARS-CoV-2 variants from these states and UT during the “Term2”. In “MH”, more than 50% of the sequences had seven co-mutations. “KA” also has a similar trend. However, for states like WB and GJ, the maximum percentage of sequences contains less than five co-mutations per sequence (Figure 3I). Figures 4A–C show networks of co-mutations for TG, MH, and WB for “Term 3”. Interestingly, in “Term3”, a higher number of co-mutations was observed in sequences retrieved from both MH and TG (Figure 4D), maintaining the trend observed in “Term2”. For example, in TG, more than 70% of the sequences harbored eight and above mutations within a single viral genome, whereas in MH close to 45% sequences possessed seven co-mutations. WB continued to harbor a lower number of co-mutations even in “Term3”, with almost 60% of the sequences having two and three co-mutations per viral sequence.

### Dynamics of Co-mutation Patterns in Indian States Across “Term2” and “Term3”

**FIGURE 5:**
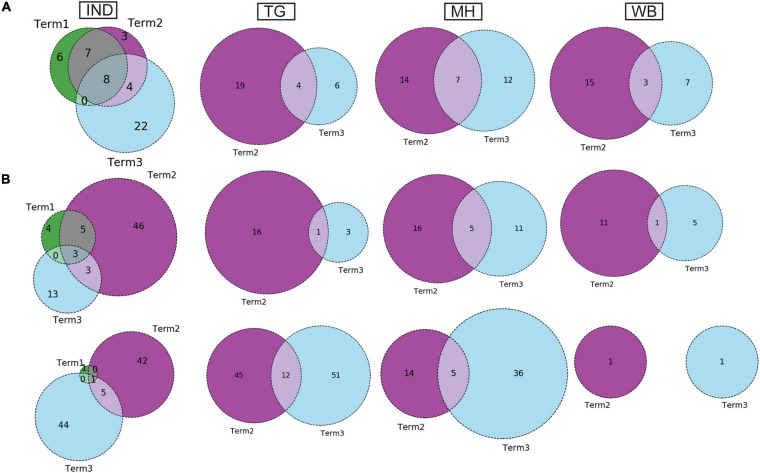
Dynamics of mutation and co-mutation pattern in Indian states across time spans. **(A)** The overlap of frequent mutations observed in overall Indian samples (IND), Telangana (TG), Maharashtra (MH), and West Bengal (WB), respectively, collected across Term1 (green), Term2 (purple), and Term3 (cyan). **(B)** The overlap of co-mutation patterns in three “Terms” where the upper panel represents an overlap of co-mutations having mutations between ≥3 and ≤5 sequences. The lower panel represents an overlap for >5 co-mutations per sequence.

Similar to the comparison of frequent mutations across time scale, we decided to check whether the co-mutation patterns of SARS-CoV-2 variant also varies over time within the states and union territories of India. However, as only three states possessed more than 50 sequences, we could compare the changes in co-mutation patterns/motifs for these three states only along with the overall Indian sequences. Figure 5A compares the frequent mutations across three or two terms for the sequences retrieved from India, Maharashtra (MH), Telangana (TG), and West Bengal (WB), respectively. As indicated before, a significant fraction of the novel mutations were evolved during “Term3” compared to “Term2” in India as well as in these three states. A comparison between the co-mutation networks consisting of three to five (≥3 and ≤5; Figure 5B upper panels) and more than five mutations (>5; Figure 5B, lower panels) again shows the emergence of a large number of unique co-mutation patterns and combinations for overall Indian sequences as well as sequences retrieved from MH, TG, and WB, respectively. This observation is further corroborated when we compared the co-mutation pattern within the major clades (G, GH, and GR, respectively) (Supplementary Figure 5). GR clade has a large number of co-occurred mutations of size (*n*) > 5 in “Term3” (Supplementary Figure 5B), which is consistent with the large number of co-occurred mutations of size (*n*) > 5 in MH and TG states (Figure 4D) where GR is the dominant clade (Supplementary Figure 4A).

### Association of Specific Mutations and Co-mutations With Patient Status

**FIGURE 6:**
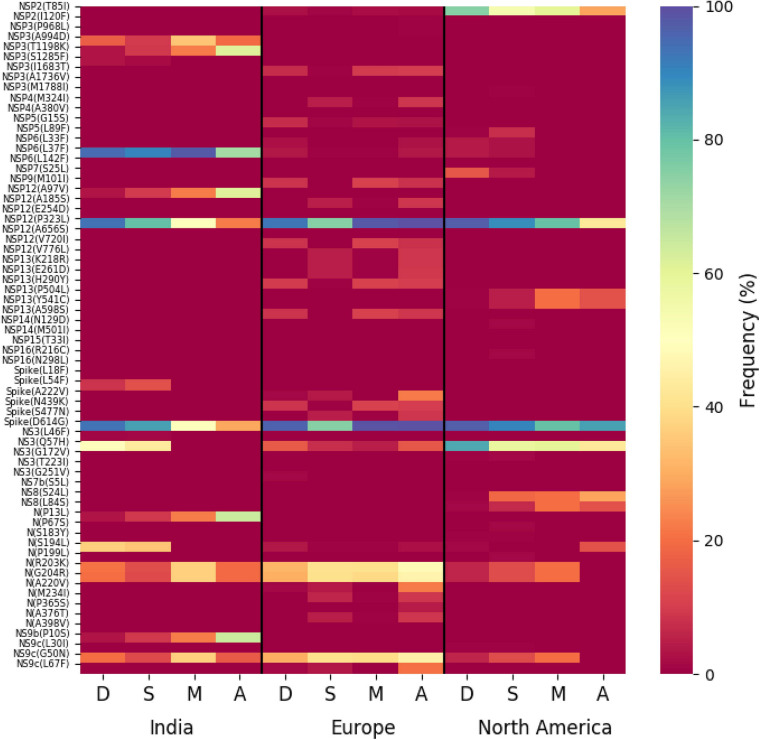
Association of specific mutations with the status of the COVID-19 patients from India, Europe, and North America. A heat map of mutations and their frequencies that were found to be associated with four different categories of the status of the COVID-19 patients—deceased (D), symptomatic (S), mild (M), and asymptomatic (A), respectively—is shown.

The association of prevalent mutations with the status of the infected patients is a key aspect to explore the connection between genetic variability and the patho-physiology of the COVID-19 disease. Hence, we calculated the fraction of mutations that are predominantly found in a subset of Indian COVID-19 patients and as well as patients from different continents for which disease outcome information is available at the GISAID database. Out of the 3,277 sequences deposited from India during 2020, patient status was reported for only 806 sequences, where 95 were marked as deceased and 631, 49, and 31 were marked as symptomatic, mild, and asymptomatic, respectively. Figure 6 provides a matrix representation of the percentage of frequent mutations observed within the Indian patients marked as deceased (D), symptomatic (S), mild symptomatic (M), and asymptomatic (A), respectively. Since the number of samples with patient status was small, the statistical significance of the association of these mutations with respective categories was evaluated using Fisher’s exact test (see section “Materials and Methods” for details), and the list of mutations that are found to be significant (*p*-value ≤ 0.05) is presented in Supplementary Table 2. It is evident that there are some mutations [NS3(Q57H), N(S194L), and Spike(L54F)] which are specifically associated with symptomatic and deceased status apart from the usual Spike(D614G) and NSP12(P323L) mutations (Figure 6). Among them, NS3(Q57H), marker for GH clade, is associated with deceased and symptomatic patients in North America where GH is also the dominant clade (Supplementary Figure 2). NSP2(T85I) is also associated with deceased and symptomatic patients in North America but not in India. However, there are also some mutations [NS9b(P10S), N(P13L), NSP12(A97V), and NSP3(T1198K), respectively] that are relatively more specifically associated with asymptomatic patients in India. Very few such asymptomatic-specific mutations apart from N(S194L) were observed in North American frequent mutations (Figure 6). Moreover, 15.54% of the deceased population and 3.91% of the symptomatic population of North American patients showed NSP7(S25L) mutation, while this was missing in the mild and asymptomatic populations. In Europe, a few frequent mutations like Spike(A222V), N(A220V), and NS9c(L67F) were observed relatively more in asymptomatic patients (Figure 6). The examination of the association of clade-specific frequent mutations with the disease/patient status does not reveal much specific association. However, a relatively higher (≥10%) association was observed for a G clade (Indian population) mutation, N(S194L), which was exclusively found in 16% of symptomatic patients only (Supplementary Figure 6A). In the case of GH clade, N(S194L) is observed in more than 70% of the samples of deceased and symptomatic patients. Similarly, NSP14(T372I), a GH-clade-specific mutation (Indian population), was exclusively observed in symptomatic patients with a higher frequency (10%), but not in the deceased population. NSP2(T85I) does not show any specific association with any patient status in overall European patients (Figure 6); however, within GH clade, it is reported in 50% mild patients (Supplementary Figure 6B). The European GR-clade-specific NSP3(K945N), Spike(L5F), Spike(S549Y), Spike(M1229I), and NS9b(R13L) mutations show more than 10% abundance in mild patients only (Supplementary Figure 6B). NSP13(S485L), NSP14(T250I), and NS3(S74F) from GH clade, NSP3(P340S), NSP3(I414V), NSP13(A505V), NS3(W131C), and N(D377Y) from GR clade, and NSP3(T1456I), NSP4(T492I), NSP14(T113I), Spike(A262S), Spike(P272L), Spike(G639S), NS7a(Q94L), NS8(I121L), and N(P365S) from GV clades seem to be associated with asymptomatic patients only (Supplementary Figure 6B). In the North American GH-clade-specific frequent mutation, NSP14(A320V) was found exclusively only in deceased patients (16%), whereas NSP5(L89F) and NS8(S24L) were observed in 14 and 32% symptomatic patients, respectively (Supplementary Figure 6C).

**FIGURE 7:**
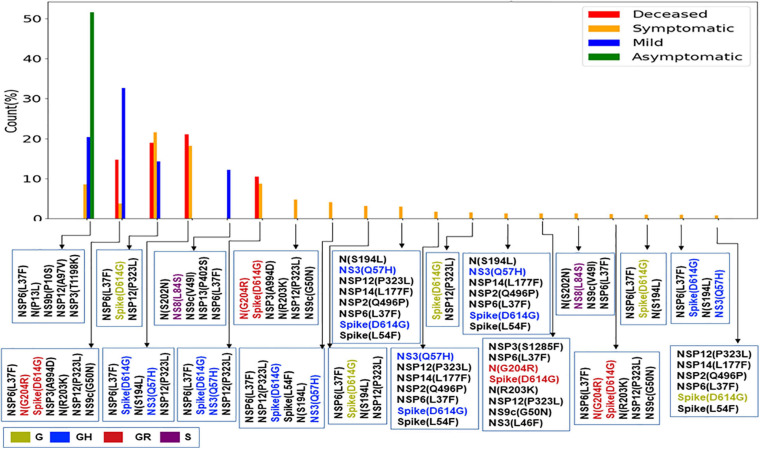
Association of specific co-mutation patterns with the status of the COVID-19 patients from India. The frequencies of specific co-mutations are plotted with respect to the four status categories of COVID-19 patients: deceased, symptomatic, mild, and asymptomatic, respectively. G, GH, GR, and S clades defining mutations are marked in yellow, blue, red, and purple, respectively.

The impact of the single-point mutation could be essential but may not be sufficient to elicit a pathological response and/or variation in disease phenotype. Hence, we thought of exploring the association of evolutionary selected multiple mutations within a viral genome with respect to the disease status of the patient from whom the sample was isolated. Figure 7 presents the frequency of patients infected with SARS-CoV-2 variants that harbor the co-mutation combinations. A combination of five co-mutations [NSP3(T1198K)-NSP6(L37F)-NSP12(A97V)-NS9b(P10S)] was found to be significantly higher (>50%) in asymptomatic patients, whereas the co-mutations of NSP3(A994D)-NSP6(L37F)-NSP12(P323L)-Spike(D614G)-N(G204R)-N(R203K)-NS9c(G50N) and NSP6(L37F)-NSP12(P323L)-Spike(D614G)-NS3(Q57H)-N(S1 94L) were observed in higher frequencies in mild patients (Figure 7). However, it is evident that the co-mutation of NSP6(L37F)-NSP12(P323L)-Spike(D614G) is present as either triad or part of a larger co-mutation network in most of the symptomatic patients along with mild, symptomatic, and deceased populations. However, quite different co-mutation patterns are observed with patients from Europe and North America, where the absence of NSP6(L37F) mutation was along with Spike(D614G) and NSP12(P323L) mutations (Supplementary Figure 7).

## Discussion

The whole-genome sequencing of SARS-CoV-2 variants throughout the world has provided an enormous amount of knowledge about the evolutionary diversity of this deadly virus and also contributed significantly in understanding the nature of the pandemic (Kim et al., 2020; Zhang and Holmes, 2020). During the difficult times in late 2019 and throughout 2020, sequencing of the viral samples was one of the primary research objectives globally in order to understand the specific phylogenetic variations and their connection to the spread and transmissibility of the virus (Wang et al., 2021). As part of this global endeavor, the deposition of SARS-CoV-2 genome data in genomic databases such as GenBank (Benson et al., 2013), NCBI (SARS-CoV-2 NCBI, 2021), and GISAID (GISAID, 2021) has truly facilitated the knowledge to tackle the deadly COVID-19 disease. Here 54 SARS-CoV-2 whole genomes have been sequenced using NGS platforms with very high coverage and depth (Supplementary Figure 1B). The viral RNA was extracted from patients reported in a hospital of Kolkata, capital of West Bengal state of India. This relatively smaller dataset of 54 RNAs was collected during the time period of August–October 2020 and constituted almost all the SARS-CoV-2 (except one) genome sequences deposited from West Bengal in the latter half of the year 2020, as was available in GISAID on January 1, 2021. However, the clade distributions of these 54 sequences constituting the “Term3” repertoire of WB sequences are distinctively different from that of overall India (Supplementary Figure 1 and Figure 1). WB “Term3” sequences have significantly higher fractions of “O” clade (GISAID) and 20A clade (Nextstrain) sequences compared to both the overall Indian distribution and the sequences retrieved during the “Term3” period as well. As a matter of fact, the “Term2” (April 2020–July 2020) sequences from WB show a significantly higher fraction of “G” and 20A clade distribution (82 and 83%, respectively) compared to other states and overall India (Figures 1, 2). This indicates that the evolutionary dynamics of the WB variants are slower and therefore may be the usual fixation of major clades like “GR” or 20B which is delayed for some reason. The number of sequences deposited from West Bengal in “Term2” and “Term3” is relatively lesser (∼200), and therefore the difference in clade distributions may lack statistical confidence. Hence, it would be rather speculative to postulate the reasons behind the apparent slower evolutionary dynamics of the viral population prevalent within West Bengal during the second half of 2020.

Intrigued by this observation, we thought that it would be helpful to undertake an analysis to examine the variations in evolutionary dynamics of the SARS-CoV-2 virus and its connection to the spatio-temporal regulation. In other words, we thought that it would be worthwhile to check the evolutionary changes in viral populations prevalent in India across time and geographical zones. Our analysis clearly shows the gradual fixation of the more prevalent clades such as GR (GISAID) and 20B (Nextstrain) across time within the viral population extracted from Indian patients. However, the most frequent mutations observed within the population across three time terms were quite different, and the emergence of newer mutations was observed even at the end of the year 2020 for the overall Indian population as well as the populations categorized based on their clades. Similarly, sequences belonging to same clades but extracted from different states also show little commonality in the type of non-clade-defining mutations (Figures 2, 3 and Table 2).

Furthermore, we also examined the variation in the co-occurrence of mutations (co-mutation) across Indian states and union territories compared over the three time terms. We observed a large number of non-clade-defining mutations to co-occur within SARS-CoV-2 variants from the states and union territories during “Term2” and “Term3”, which is consistent with earlier reports based on the sequences collected mostly in “Term1” period (Sarkar et al., 2021). Consequently, a larger number of unique combinations among these co-occurred mutations appeared during the latter half of the year 2020. It is really interesting to observe that larger fractions of viral samples extracted from states like Telangana, Maharashtra, and Karnataka during “Term2” harbored five or more co-mutations, while major fractions from West Bengal and Gujarat possessed less than five co-mutations. Even in “Term3”, far larger fractions of sequences from Telangana and Maharashtra showed higher co-mutating residues compared to that of West Bengal. Significantly higher numbers of active infection cases were reported in Maharashtra and Karnataka compared to West Bengal and Gujarat during “Term2” and “Term3” (Pattabiraman et al., 2020; COVID19india, 2021). Hence, the higher number co-mutations in those two states could be associated with the higher infectivity of the variants prevalent there. In West Bengal, during “Term3”, only ∼22% sequences harbored more than five co-mutations, while almost 58% of the sequences were designated as “O” clade known for harboring a larger amount of other (Mercatelli and Giorgi, 2020) mutations.

**FIGURE 8:**
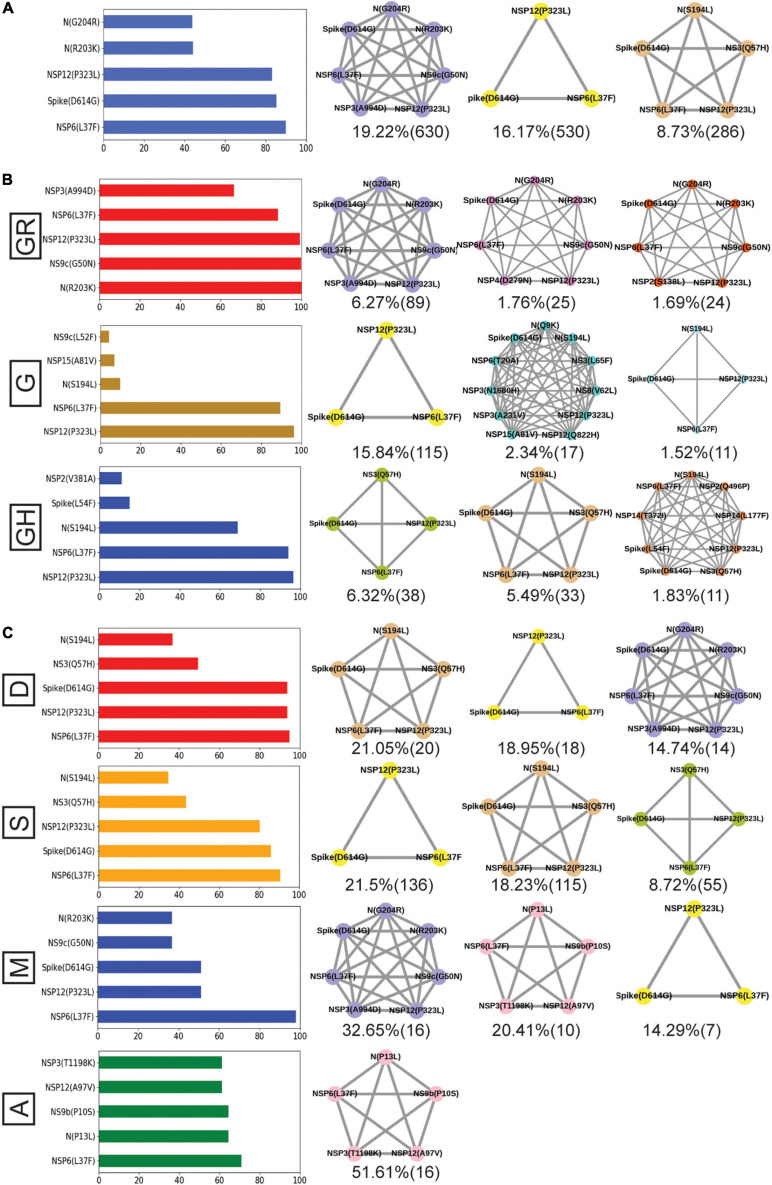
Frequent mutations, co-mutations, and their association with disease severity in India. **(A)** The most frequent five mutations and three co-mutation patterns in India. **(B)** The most frequent five mutations and three co-mutations in the major clades of India. Clade-defining mutations are excluded in the bar plot. **(C)** The most frequent five mutations and three co-mutations in different types of patients. Different color codes are used for specific co-mutation network motifs. The network motifs were sorted and ranked exclusively.

Finally, we tried to associate the frequent mutations and co-mutation patterns along with COVID-19 patient status broadly categorized into deceased, symptomatic, mild, and asymptomatic groups, respectively. Although the number of patient status mapped data is lower (806 out of 3,277 sequences), we thought that it was worthwhile to investigate if some of the mutations and co-mutation patterns could be specifically associated with those four states of the COVID-19 disease. Apart from the usual Spike(D614G) and NSP12(P323L) mutations, a specific association of mutations NS3(Q57H), N(S194L), and Spike(L54F) was observed with symptomatic and deceased status of Indian patients, while NS9b(P10S), N(P13L), NSP12(A97V), and NSP3(T1198K), respectively, are found to be relatively more in asymptomatic Indian patients. Similarly, the presence and absence of a specific mutation with respect to the symptomatic or asymptomatic status were also examined for North American and European samples for which the disease status was available at the GISAID database. Although we did not find any specific co-mutation pattern with severe (deceased or symptomatic) patients, a few co-mutation patterns were observed to be significantly higher in asymptomatic and mild symptomatic patients (Figure 7). We have also identified the presence of a co-mutation triad [NSP6(L37F)-NSP12(P323L)-Spike(D614G)] in most of the symptomatic patients, including mild, symptomatic, and deceased populations of Indian patients. Figure 8 summarizes the most frequent mutations and co-mutation network motifs within overall Indian SARS-CoV-2 genomes, most prevalent clades, and also for the viral samples collected from patients for which the disease status was documented. We observed that the top five mutations for deceased and symptomatic patients remain identical. Among these five mutations, three appear in mild and only one mutation [NSP6(L37F)] appears in asymptomatic patients. It indicates the possibility of association of specific mutations with disease severity. A similar overlap is observed in the co-mutation networks of deceased and symptomatic patients. The co-mutation network observed in 51% of asymptomatic patients is also observed in 20.41% of mild patients but neither in deceased nor in the case of symptomatic patients. However, it must be noted that the mere association of specific viral mutations may not be sufficient to describe the cause of death. The death of the patient is intricately related to the host immune response, co-morbidity status, nature of treatment, and many other factors.

**FIGURE 9:**
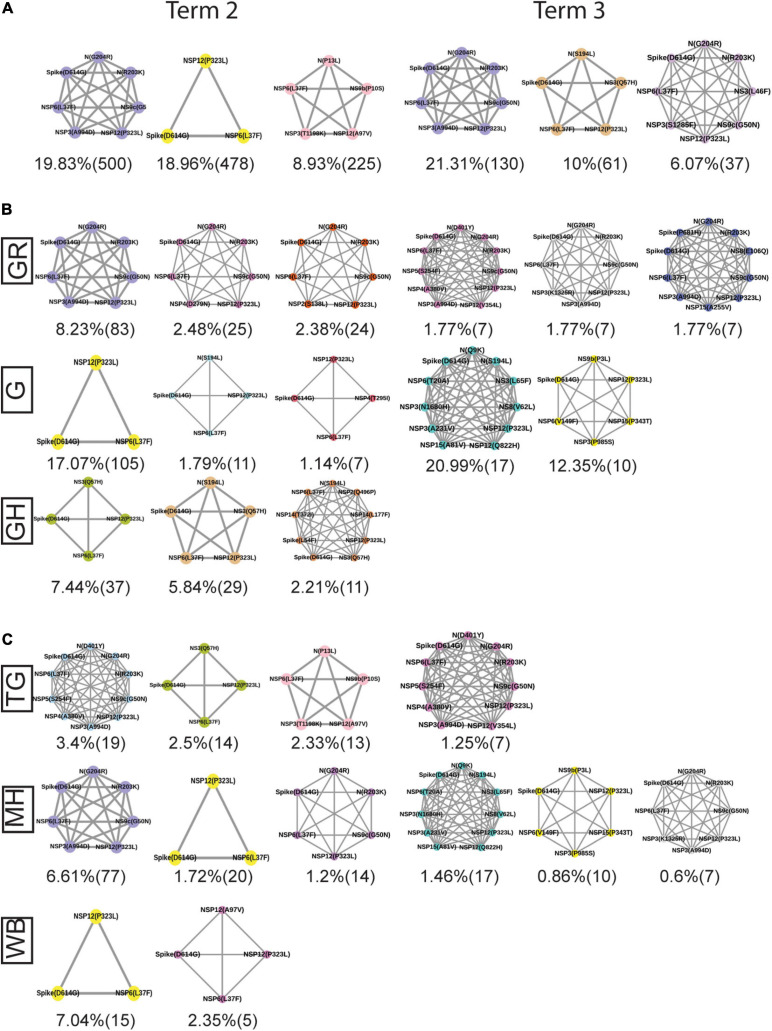
Spatio-temporal variation in the most frequent co-mutations in India. **(A)** The most frequent three co-mutations in Term2 and Term3 in India. **(B)** The most frequent three co-mutations in Term2 and Term3 in the major clades of India. **(C)** The most frequent three co-mutations in Term2 and Term3 in the different states of India. The co-mutation network observed in at least five patients are represented. Different color codes are used for specific co-mutation network motifs. The network motifs were sorted and ranked exclusively.

Figure 9 provides an overview of the dynamics of the co-mutation pattern/motif observed within viral samples from the Indian population, most prevalent clades, and for the three states (MH, TG, and WB) for which sufficient sequences were deposited in GISAID database in Term2 and Term3. It highlights the emergence of new co-mutation network motifs with time across different states and clades. In principle, the emergence of newer mutation and co-mutation patterns could influence the infectivity and transmissibility of the virus. As a matter of fact, the experience of a second wave of COVID-19 infections in India during April 2021–June 2021 where the infection rate was found to be far higher than before may indulge us to connect the previous findings to this. However, it must be noted that we did not find any of the recently observed highly transmissible variants of concerns (VOC) from the Indian cohort within the data deposited at the GISAID database till December 2020. Similarly, the emergence of more mutation and co-mutation patterns including the Spike and non-Spike mutations within the existing SARS-CoV-2 variants might as well impact the effectivity of the proposed vaccines in India (Folegatti et al., 2020; Baraniuk, 2021; Bian et al., 2021; Ella et al., 2021). However, it would be too speculative to comment on the probable impacts of the frequent mutations presented in this study with the vaccine effectivity in the absence of in-depth experimental and clinical evidence. Nevertheless, we believe that our report is one of the few studies that aim to provide a comprehensive picture of the evolutionary dynamics and co-mutation patterns of the SARS-CoV-2 variants prevalent within the Indian population throughout the year 2020. It would not be an extrapolation to suggest that our findings transcend the time scale of 2020 and serve as a harbinger of the re-emergence of the pandemic and the associated dynamic nature of the virus in 2021.

## Data Availability Statement

The datasets presented in this study can be found in online repositories. The names of the repository/repositories and accession number(s) can be found in the article/Supplementary Material.

## Ethics Statement

The studies involving human participants were reviewed and approved by Ethical Committee of CSIR-Indian Institute of Chemical Biology and Ethical Committee of Medica Superspeciality Hospital. Written informed consent for participation was not required for this study in accordance with the national legislation and the institutional requirements.

## Author Contributions

NB performed all the sequence analysis and mutational and co-mutational analyses. PM and SM sequenced the viral genomes. DB assembled the raw sequences and performed computational analysis. SS, AR, and AM provided clinical guidance, performed RNA processing, and detection of COVID-19 from bio specimens. SC, SP, and PC conceptualized and coordinated the project. NB and SC wrote the manuscript. All authors contributed to the article and approved the submitted version.

## Conflict of Interest

The authors declare that the research was conducted in the absence of any commercial or financial relationships that could be construed as a potential conflict of interest.

## Publisher’s Note

All claims expressed in this article are solely those of the authors and do not necessarily represent those of their affiliated organizations, or those of the publisher, the editors and the reviewers. Any product that may be evaluated in this article, or claim that may be made by its manufacturer, is not guaranteed or endorsed by the publisher.

## Supplementary Material

Supplementary Figure 1 Quality, demographic, and phylogenic analysis of the SARS-CoV-2 genome sequences. **(A)** Comparison of the age distribution of the patients from which the 54 SARS-CoV-2 sequenced variants (IICB) were extracted with respect to the overall age distribution of the Indian COVID-19 patients (India). **(B)** The depth (shown as multiples of “X”) of the sequenced data for both long (Nanopore) and short (Illumina) read sequences. **(C–F)** Plots of the GISAID (left) and Nextstrain (right) clade distribution of the 54 genomes sequenced by CSIR-IICB and MEDICA Superspecialty Hospital and deposited from India overall, respectively.

Supplementary Figure 2 Distribution and dynamics of SARS-CoV-2 clades in the world for three different time spans in the year 2020. **(A–C)** Distribution of GISAID clades across the world for three different times spans: “Term1” (December 2019–March 2020), “Term2” (April 2020–July 2020), and “Term3” (August 2020–December 2020), respectively.

Supplementary Figure 3 Overlap of frequent mutations across three terms for the most prevalent GISAID and Nextstrain clades in India. **(A)** The overlap of frequent mutations observed in the most prevalent GISAID clades like G, GH, and GR collected across Term1 (green), Term2 (purple), and Term3 (cyan), respectively. **(B)** The overlap of frequent mutations observed in the most prevalent Nextstrian clades like 19A, 20A, and 20B collected across Term1 (green), Term2 (purple), and Term3 (cyan), respectively.

Supplementary Figure 4 Distribution of GISAID clades within Indian states and comparison of frequent mutations across them during Term3 (August 2020–December 2020). **(A)** The distribution of GISAID clades in three Indian states that deposited more than 50 SARS-CoV-2 sequences during August 2020–December 2020. **(B–D)** The number of sequences and frequent mutations of the three states and the number of common mutations among them for G, GH, and GR clades, respectively.

Supplementary Figure 5 Dynamics of the co-mutation pattern across three terms for the most prevalent GISAID clades in India. The overlap of co-mutation patterns in three “Terms” for co-mutations having mutations between ≥3 and ≤5 sequences **(A)** and for >5 co-mutations per sequence **(B)** is shown.

Supplementary Figure 6 Association of specific mutations with the status of the COVID-19 patients from India, Europe, and North America infected with specific clades of the SRAS-CoV-2 virus. A heat map of mutations and their frequencies that were found to be associated with four different categories of the status of COVID-19 patients—deceased (D), symptomatic (S), mild (M), and asymptomatic (A), respectively—is shown for the most prevalent clades from India **(A)**, Europe **(B)**, and North America **(C)**, respectively.

Supplementary Figure 7 Association of specific co-mutation patterns with the status of the COVID-19 patients from Europe and North America. Frequencies of specific co-mutations are plotted with respect to the four status categories of COVID-19 patients: deceased, symptomatic, mild, and asymptomatic, respectively. **(A)** Data from Europe. **(B)** Data from North America. Clade-defining mutations are marked in corresponding colors.
