# Corrigendum: Genomic surveillance and phylodynamic analyses reveal the emergence of novel mutations and co-mutation patterns within SARS-CoV-2 variants prevalent in India

**DOI:** 10.3389/fmicb.2024.1444349

**Published:** 2024-07-08

**Authors:** Nupur Biswas, Priyanka Mallick, Sujay Krishna Maity, Debaleena Bhowmik, Arpita Ghosh Mitra, Soumen Saha, Aviral Roy, Partha Chakrabarti, Sandip Paul, Saikat Chakrabarti

**Affiliations:** ^1^Structural Biology and Bioinformatics Division, Council for Scientific and Industrial Research (CSIR) - Indian Institute of Chemical Biology (IICB), Kolkata, India; ^2^Academy of Scientific and Innovative Research (AcSIR), Ghaziabad- 201002, India; ^3^Cell Biology & Physiology Division, Council for Scientific and Industrial Research (CSIR) - Indian Institute of Chemical Biology (IICB), Kolkata, India; ^4^MEDICA Superspecialty Hospital, Kolkata, India

**Keywords:** genome sequencing, India, mutation – genetics, phylodynamic analyses, SARS-CoV-2, COVID-19

In the published article, there was an error in affiliation 2. Instead of “Academy of Scientific and Innovative Research (AcSIR), Ghaziabad, India,” it should be “Academy of Scientific and Innovative Research (AcSIR), Ghaziabad- 201002, India.”

The authors apologize for this error and state that this does not change the scientific conclusions of the article in any way. The original article has been updated.

