# Supplementary figures and images for: Genomic Surveillance and Phylodynamic Analyses Reveal the Emergence of Novel Mutations and Co-mutation Patterns Within SARS-CoV-2 Variants Prevalent in India
Source: Front Microbiol. 2021 Jul 29;12:703933. doi: 10.3389/fmicb.2021.703933 (PMC8358439; doi:10.3389/fmicb.2021.703933)

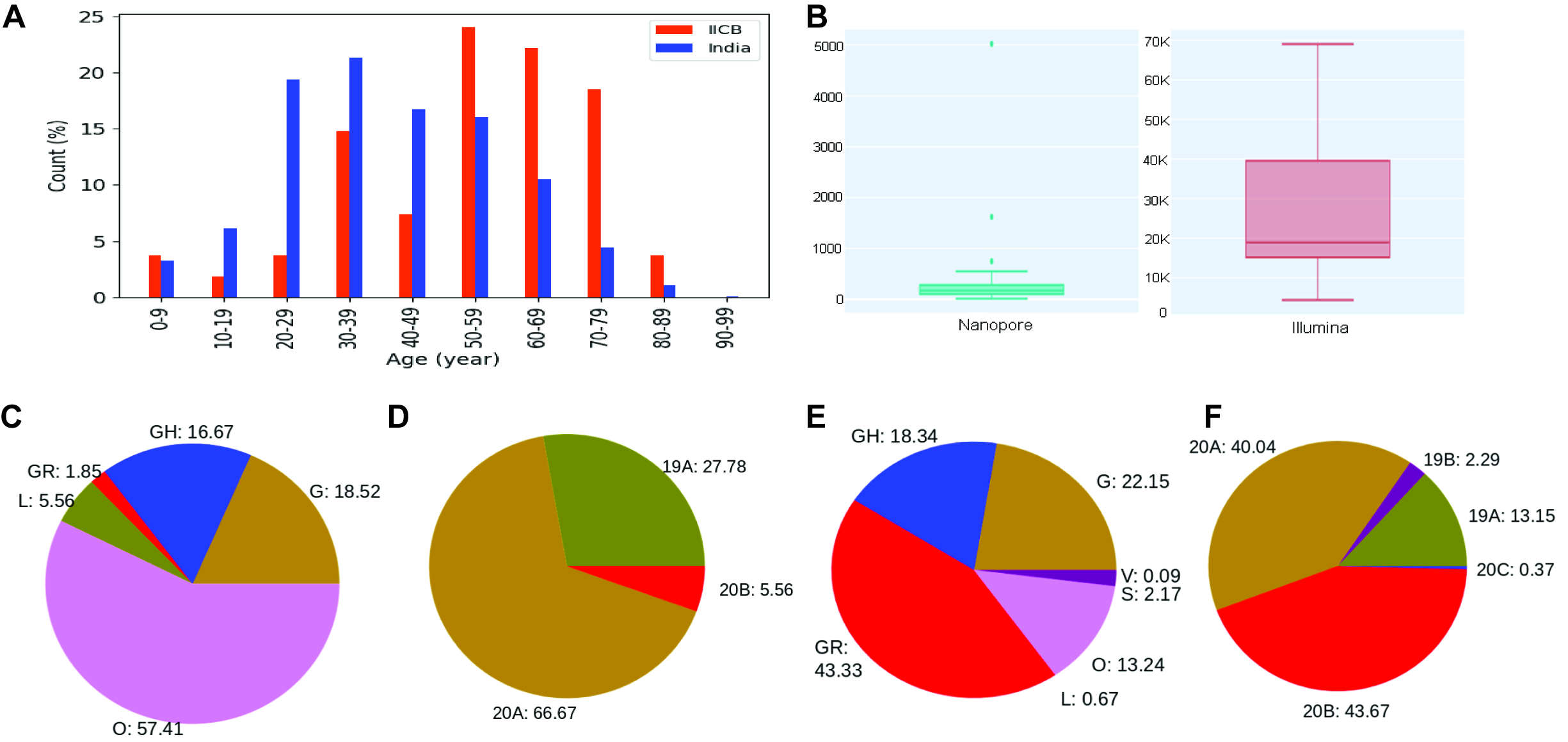

Supplement: Supplementary Figure 1 — Quality, demographic, and phylogenic analysis of the SARS-CoV-2 genome sequences. (A) Comparison of the age distribution of the patients from which the 54 SARS-CoV-2 sequenced variants (IICB) were extracted with respect to the overall age distribution of the Indian COVID-19 patients (India). (B) The depth (shown as multiples of “X”) of the sequenced data for both long (Nanopore) and short (Illumina) read sequences. (C–F) Plots of the GISAID (left) and Nextstrain (right) clade distribution of the 54 genomes sequenced by CSIR-IICB and MEDICA Superspecialty Hospital and deposited from India overall, respectively. [file Image_1.TIF]

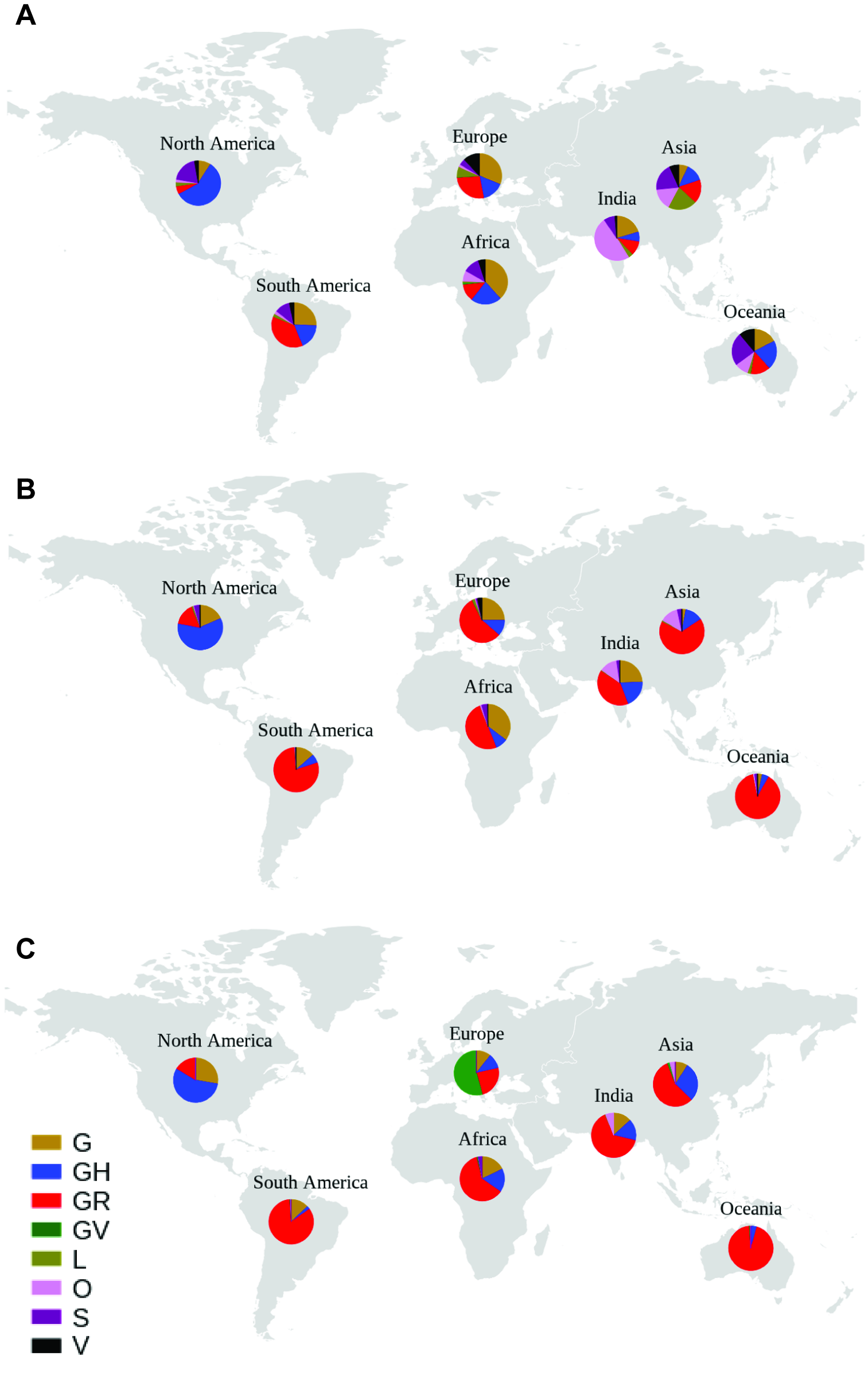

Supplement: Supplementary Figure 2 — Distribution and dynamics of SARS-CoV-2 clades in the world for three different time spans in the year 2020. (A–C) Distribution of GISAID clades across the world for three different times spans: “Term1” (December 2019–March 2020), “Term2” (April 2020–July 2020), and “Term3” (August 2020–December 2020), respectively. [file Image_2.TIF]

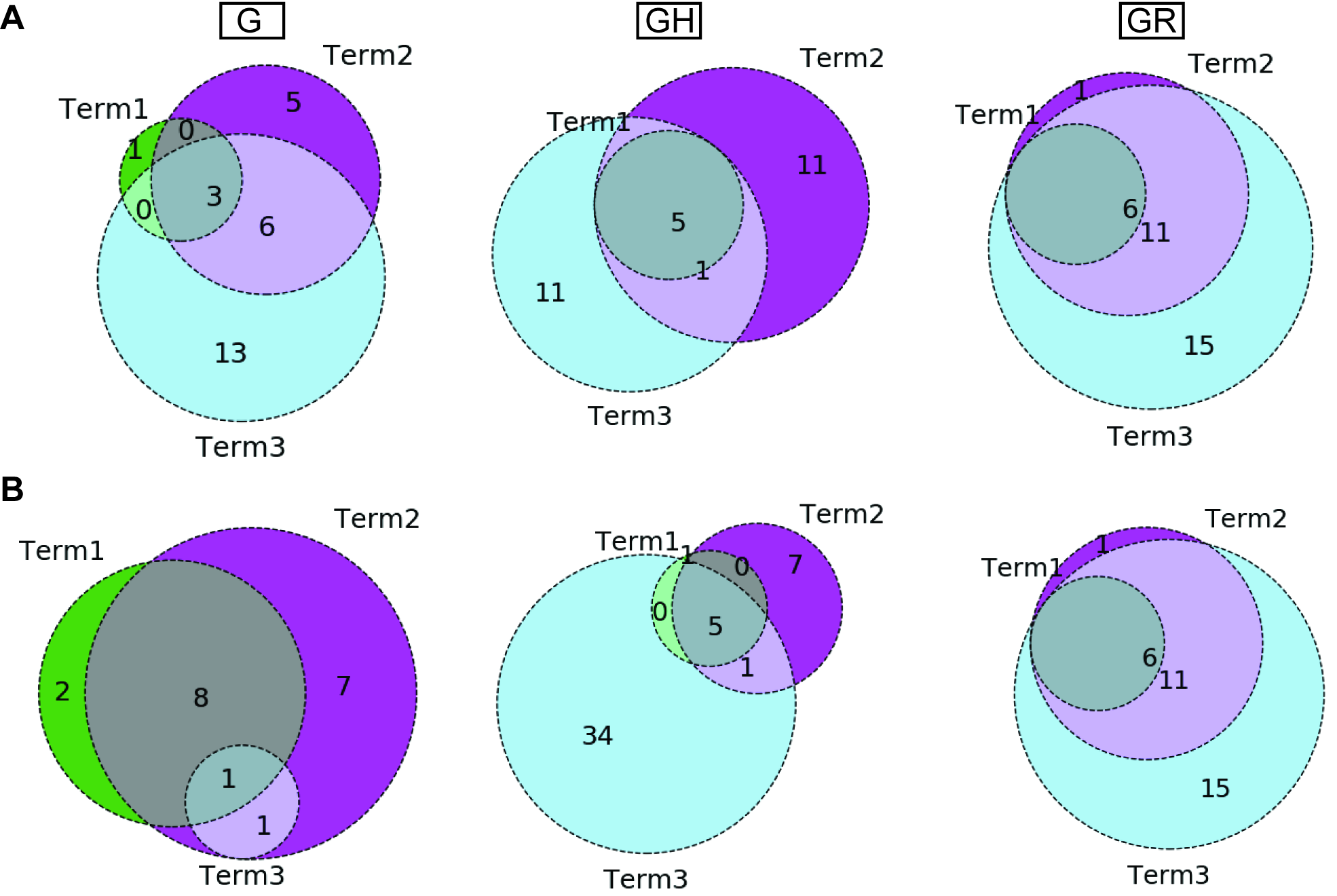

Supplement: Supplementary Figure 3 — Overlap of frequent mutations across three terms for the most prevalent GISAID and Nextstrain clades in India. (A) The overlap of frequent mutations observed in the most prevalent GISAID clades like G, GH, and GR collected across Term1 (green), Term2 (purple), and Term3 (cyan), respectively. (B) The overlap of frequent mutations observed in the most prevalent Nextstrian clades like 19A, 20A, and 20B collected across Term1 (green), Term2 (purple), and Term3 (cyan), respectively. [file Image_3.TIF]

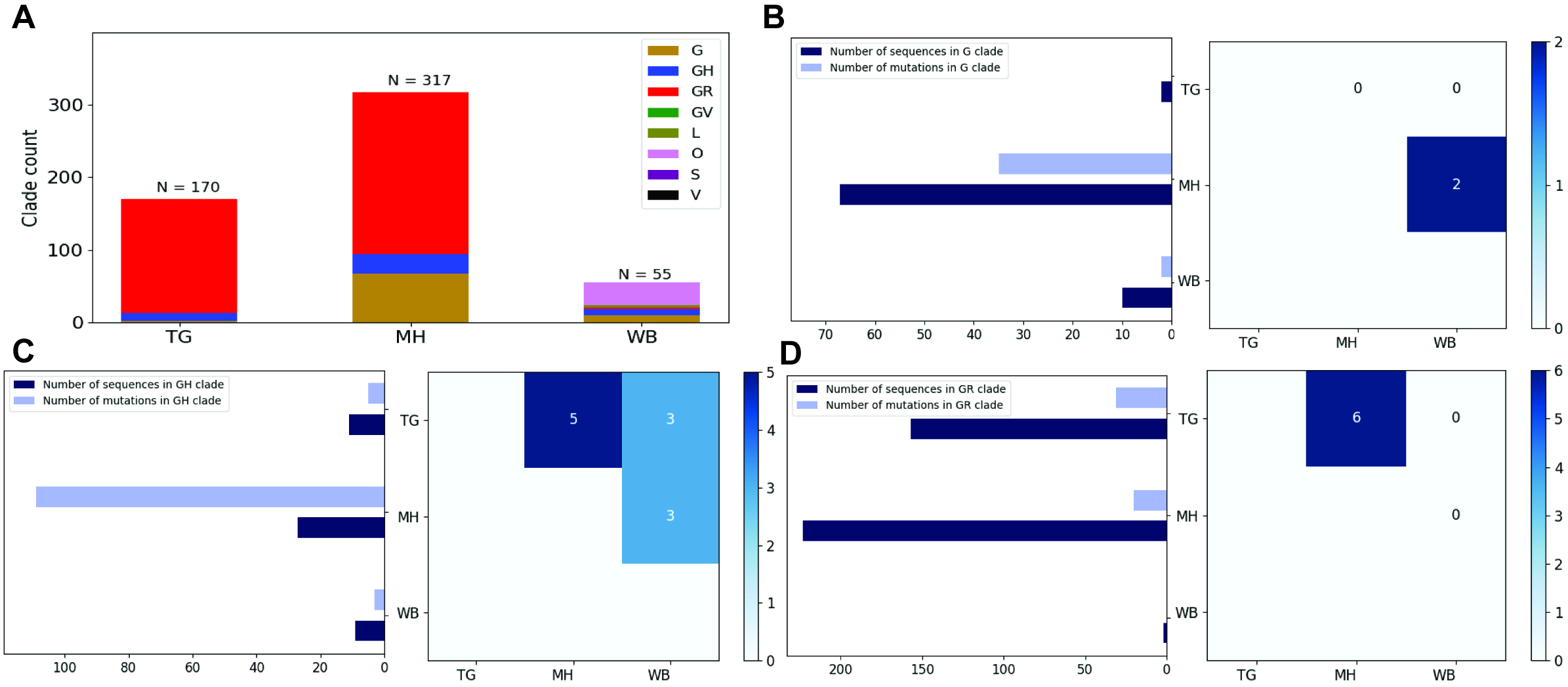

Supplement: Supplementary Figure 4 — Distribution of GISAID clades within Indian states and comparison of frequent mutations across them during Term3 (August 2020–December 2020). (A) The distribution of GISAID clades in three Indian states that deposited more than 50 SARS-CoV-2 sequences during August 2020–December 2020. (B–D) The number of sequences and frequent mutations of the three states and the number of common mutations among them for G, GH, and GR clades, respectively. [file Image_4.TIF]

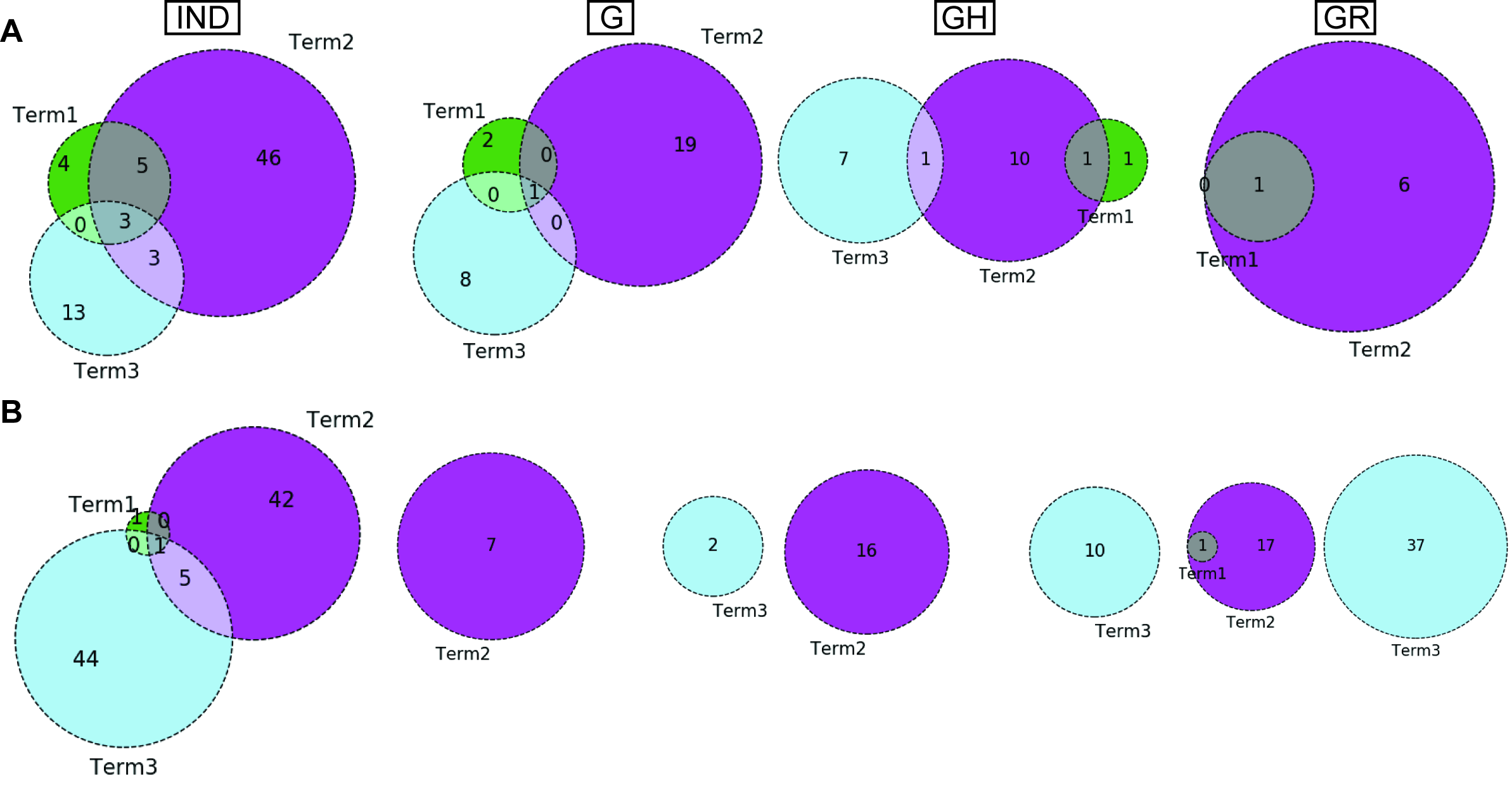

Supplement: Supplementary Figure 5 — Dynamics of the co-mutation pattern across three terms for the most prevalent GISAID clades in India. The overlap of co-mutation patterns in three “Terms” for co-mutations having mutations between ≥3 and ≤5 sequences (A) and for >5 co-mutations per sequence (B) is shown. [file Image_5.TIF]

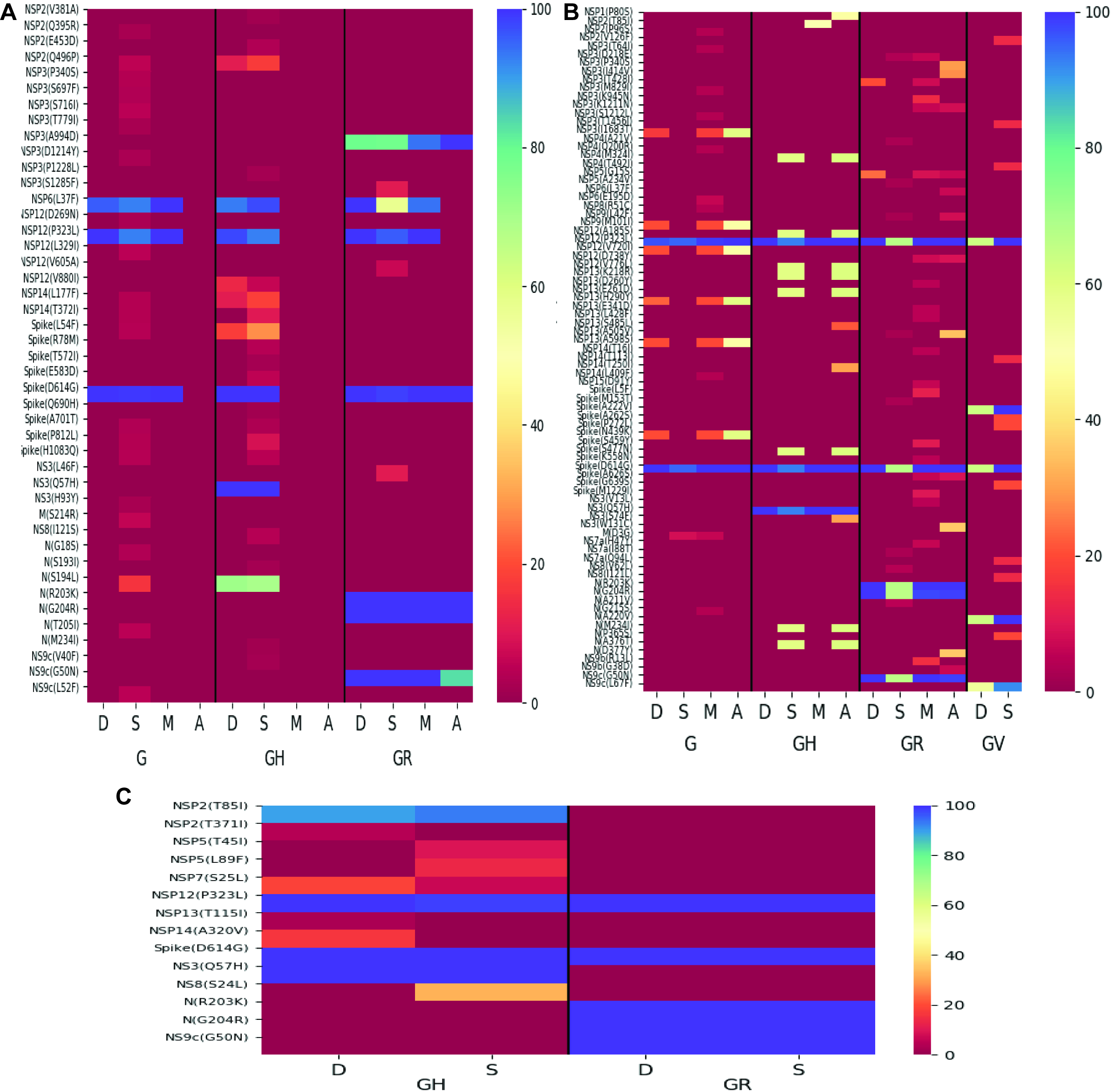

Supplement: Supplementary Figure 6 — Association of specific mutations with the status of the COVID-19 patients from India, Europe, and North America infected with specific clades of the SRAS-CoV-2 virus. A heat map of mutations and their frequencies that were found to be associated with four different categories of the status of COVID-19 patients—deceased (D), symptomatic (S), mild (M), and asymptomatic (A), respectively—is shown for the most prevalent clades from India (A), Europe (B), and North America (C), respectively. [file Image_6.TIF]

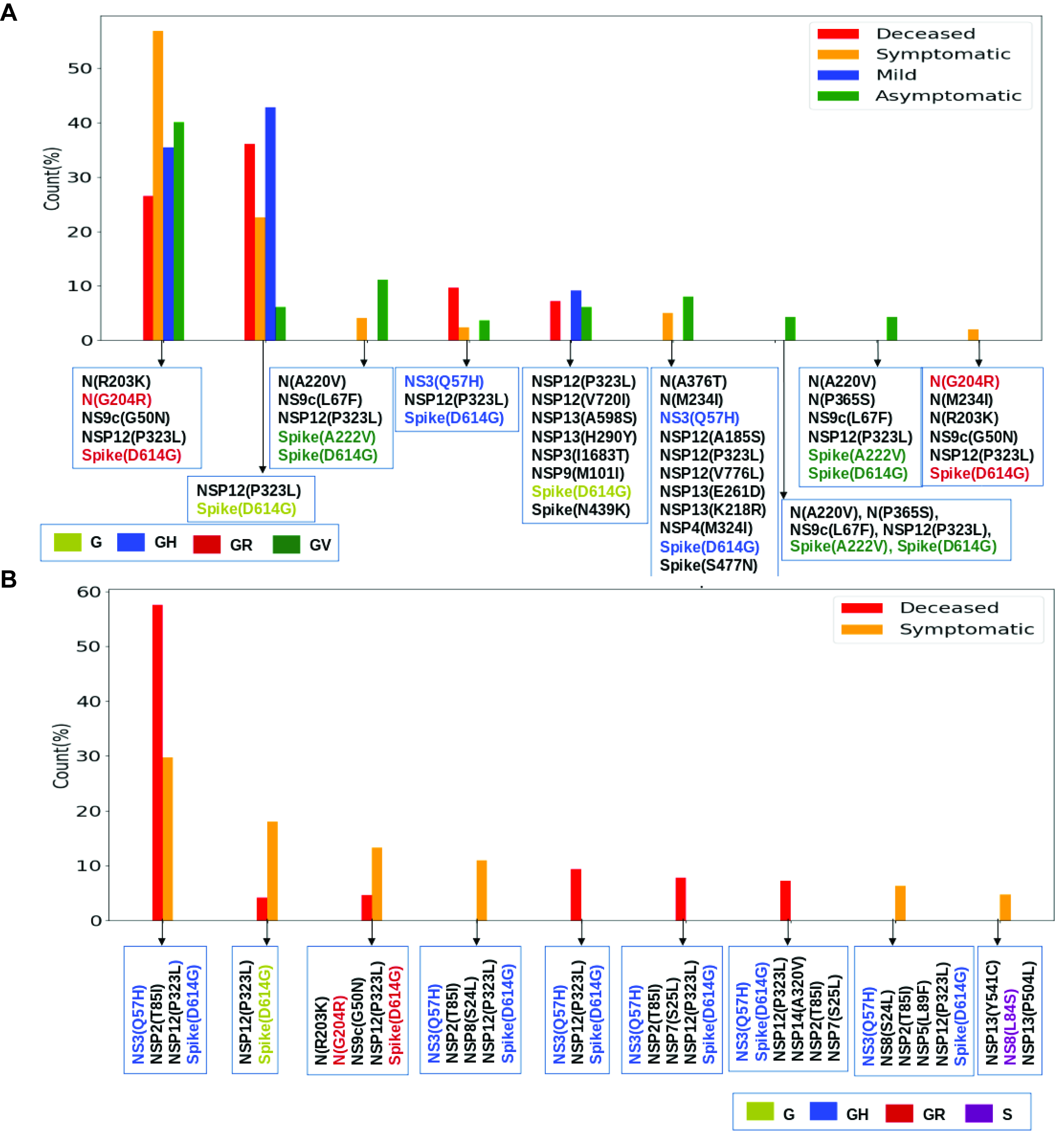

Supplement: Supplementary Figure 7 — Association of specific co-mutation patterns with the status of the COVID-19 patients from Europe and North America. Frequencies of specific co-mutations are plotted with respect to the four status categories of COVID-19 patients: deceased, symptomatic, mild, and asymptomatic, respectively. (A) Data from Europe. (B) Data from North America. Clade-defining mutations are marked in corresponding colors. [file Image_7.TIF]
